# Current Evidence Linking Microplastic Exposure and Reproductive Cancers

**DOI:** 10.3390/jox16050163

**Published:** 2026-08-31

**Authors:** Barira Rais, Abul Vafa, Faten F. Bin Dayel, Summya Rashid

**Affiliations:** 1 School of Chemical & Life Sciences, Jamia Hamdard, New Delhi 110062, India; barira933@gmail.com (B.R.); alwafakhan@gmail.com (A.V.); 2 Department of Pharmacology & Toxicology, College of Pharmacy, Prince Sattam Bin Abdul Aziz University, Al Kharj 11942, Saudi Arabia; f.bindayel@psau.edu.sa

**Keywords:** microplastics, nanoplastics, reproductive cancers, carcinogenesis, oxidative stress, genotoxicity

## Abstract

Microplastics (MPs) and nanoplastics (NPs) have emerged as pervasive environmental contaminants with increasing evidence of human exposure and biological accumulation. Recent studies have confirmed their presence in multiple human reproductive tissues and fluids, including semen, testicular tissue, ovarian follicular fluid, cervicovaginal secretions, placenta, and breast milk, raising concerns regarding their potential implications for reproductive health. Beyond their widespread distribution, MPs have been shown in experimental studies to interact with cellular and molecular processes, including oxidative stress, inflammatory responses, mitochondrial dysfunction, and DNA damage, which are pathways commonly implicated in carcinogenesis. This review provides a comprehensive and critical synthesis of current evidence linking microplastic exposure to reproductive cancers, including prostate, testicular, ovarian, endometrial, cervical, and vaginal malignancies. Available mechanistic studies suggest that MPs may influence cancer-related biological processes through dysregulation of programmed cell death, genotoxicity, endocrine disruption, and modulation of signaling pathways such as PI3K/AKT and MAPK. Experimental findings also indicate that MPs may alter the tumor microenvironment and affect cellular behaviors associated with proliferation, migration, and invasion. However, the majority of current evidence is derived from in vitro studies, animal models, and indirect mechanistic observations, while direct epidemiological evidence in humans remain limited. Furthermore, methodological heterogeneity in microplastic detection and characterization complicates comparisons across studies and hinders causal inference. Overall, current evidence supports the biological plausibility of an association between microplastic exposure and reproductive cancer-related processes, while highlighting the need for standardized methodologies and well-designed longitudinal human studies to clarify potential health risks.

## 1. Introduction

The exponential rise in global plastic production over recent decades has resulted in the widespread accumulation of plastic waste, raising significant concerns regarding its long-term environmental and human health consequences [1,2]. Due to their non-biodegradable nature, plastics undergo gradual fragmentation through physical, chemical, and biological processes, generating microplastics (MPs; <5 mm) and nanoplastics (NPs; <1 μm). These particles are now ubiquitously distributed across environmental matrices, including aquatic systems, soil, air, and food chains, making human exposure unavoidable through ingestion, inhalation, and dermal contact [3,4]. Recent analytical advancements have confirmed the presence of microplastics in multiple human biological samples, highlighting their ability to cross biological barriers and accumulate within tissues [5,6].

Recent reviews have highlighted that micro- and nanoplastics represent a growing global environmental and public health concern due to their widespread distribution across ecosystems, persistence in biological systems, and potential interactions with cellular and molecular processes relevant to chronic diseases. Increasing evidence suggests that their impacts extend beyond ecological toxicity and may include long-term effects on endocrine, reproductive, metabolic, and immune health [7,8].

Beyond their widespread occurrence, MPs are increasingly recognized as biologically active contaminants capable of interacting with cellular and molecular processes relevant to human health. Experimental studies have shown that microplastic exposure can induce oxidative stress, inflammatory responses, mitochondrial dysfunction, DNA damage, and alterations in cellular signaling pathways [9,10]. In addition, microplastics can act as carriers of toxic additives and environmental pollutants, including endocrine-disrupting chemicals (EDCs), which may further modify biological responses [11,12]. While these findings support the biological plausibility of a potential relationship between MP exposure and cancer-related processes, the majority of available evidence originates from in vitro and animal studies and does not establish a direct causal role in carcinogenesis [10].

The reproductive system is particularly vulnerable to such environmental stressors due to its high sensitivity to hormonal regulation and tightly controlled cellular processes. Recent studies have detected microplastics in a range of reproductive tissues and fluids, including semen, testicular tissue, ovarian follicular fluid, cervicovaginal secretions, placenta, and breast milk [13,14,15]. Their presence within these compartments suggests not only systemic distribution but also direct interaction with reproductive cells and microenvironments. In males, microplastic exposure has been associated with impaired sperm parameters, while in females, disruptions in ovarian function, hormonal balance, and oocyte quality have been reported [5]. Importantly, such alterations may extend beyond fertility impairment and contribute to cellular environments that have been implicated in carcinogenesis [16,17].

Current evidence can be broadly categorized into three levels. First, established findings include the detection of micro- and nanoplastics in human reproductive tissues and biological fluids, demonstrating their capacity for systemic distribution and tissue accumulation [14,18]. Second, proposed mechanisms, supported primarily by in vitro and animal studies, suggest that microplastics may influence oxidative stress, inflammation, endocrine signaling, genotoxicity, and other pathways relevant to carcinogenesis [19]. Third, the hypothesis that microplastic exposure directly causes reproductive cancers in humans remains speculative due to limited epidemiological evidence, methodological heterogeneity among studies, and the absence of longitudinal investigations capable of establishing causality. Building upon these observations, increasing attention has been directed toward the potential association between microplastic exposure and reproductive cancers, including prostate, testicular, ovarian, cervical, and uterine malignancies. These cancers are driven by complex interactions between genetic, hormonal, and environmental factors, with endocrine disruption and chronic inflammation playing central roles in their pathogenesis [20,21,22]. For instance, in prostate cancer in vitro models, exposure to polystyrene nanoplastics (PS-NPs) has been associated with alterations in ferroptosis-related pathways and inflammatory mediators alongside changes in cellular proliferation markers [23,24]. Similarly, in ovarian cancer in vitro models, MPs have been associated with enhanced invasive behavior and alterations in the tumor microenvironment, partly driven by oxidative stress and inflammatory signaling [25]. However, despite growing experimental and mechanistic evidence, epidemiological data directly linking microplastic exposure to reproductive cancers remain limited, while significant methodological and analytical challenges continue to hinder causal inference. Consequently, current evidence is best interpreted as supporting biological plausibility and mechanistic associations rather than demonstrating a direct causal relationship between microplastic exposure and reproductive cancer development.

Although numerous studies have investigated the reproductive toxicity of microplastics and several reviews have summarized their effects on fertility and endocrine function, a comprehensive synthesis focusing specifically on reproductive cancers remains lacking. Existing evidence is dispersed across experimental, clinical, and mechanistic studies, making it difficult to evaluate the strength of current evidence, identify key limitations, and assess whether the reported findings support potential associations between microplastic exposure and reproductive cancer-related processes. To our knowledge, no recent review has critically integrated evidence from human tissue studies, experimental models, mechanistic investigations, and cancer-specific findings to evaluate the current state of evidence linking microplastic exposure to reproductive cancers.

In this context, the present review critically synthesizes current evidence linking micro- and nanoplastic exposure to reproductive cancers. Specifically, it examines the occurrence and distribution of microplastics within the human reproductive system, evaluates their effects on reproductive health, discusses mechanistic evidence relevant to carcinogenesis, and assesses the strengths and limitations of available human and experimental studies. Furthermore, the review identifies key knowledge gaps and future research priorities to better understand the potential implications of microplastic exposure for reproductive oncology and public health.

## 2. Occurrence and Characterization of Microplastics in Human Reproductive Tissues

### 2.1. Definition and Classification

**Figure 1:**
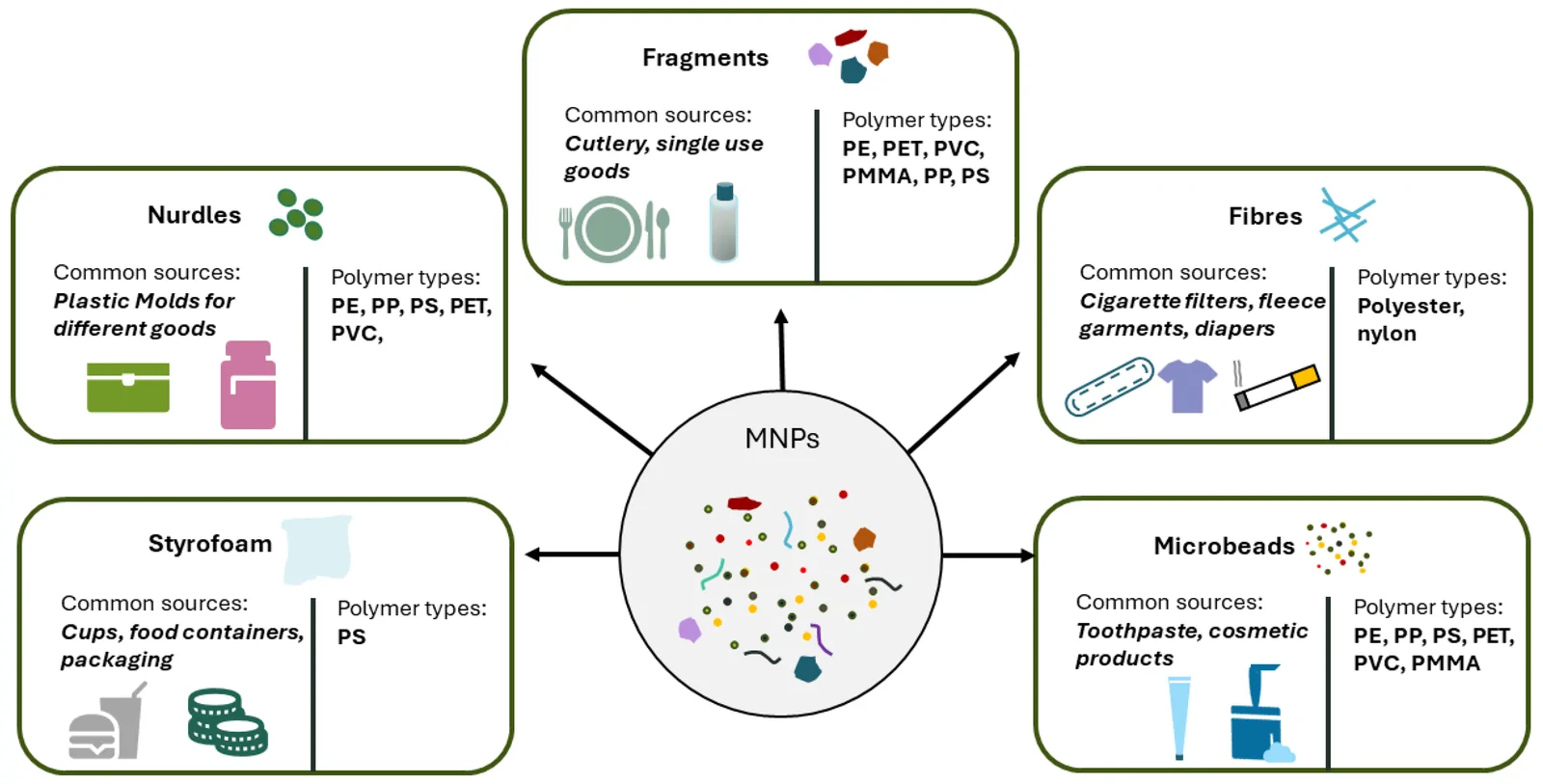
Morphological characteristics, polymer composition, and probable sources of commonly detected micro- and nanoplastics (MNPs) in human beings. PET: Polyethylene terephthalate; PS: Polystyrene; PE: Polyethylene; PP: Polypropylene; PVC: Polyvinyl chloride; PMMA: Polymethyl methacrylate.

Microplastics (MPs) are artificially produced solid polymer particles that may occur in either uniform or uneven forms, with dimensions typically ranging between 1 μm and 5 mm [26]. Nanoplastics (NPs), in contrast, are plastic particles measuring less than 1 μm in diameter and are considered particularly hazardous because their high surface area-to-volume ratio facilitates greater cellular penetration and unique toxicological behaviors [27]. Based on their origin, these particles are broadly classified as primary microplastics, which are deliberately manufactured at microscopic sizes, and secondary microplastics, which arise from the breakdown and weathering of larger plastic materials. Frequently encountered polymer categories include: polyethylene (PE), polypropylene (PP), polyvinyl chloride (PVC), polystyrene (PS), polyethylene terephthalate (PET), polyamide (PA) and polyurethane (PU), which account for the vast majority of environmental microplastic pollution, often originating from daily use items like plastic bottles, clothes, packaging, etc [28] (Figure 1).

Among these polymers, PS is the most frequently investigated polymer in experimental microplastic toxicology studies because it is commercially available in highly standardized particle sizes, possesses well-characterized physicochemical properties, and can be readily modified for laboratory exposure experiments [29,30,31]. However, PS represents only a relatively small fraction of global plastic production. According to recent market estimates, PE and PP together account for approximately 45–55% of global plastic production, whereas PS contributes only about 5–7%. Nevertheless, PS particles are commonly detected in environmental samples and biological studies due to their widespread use in food packaging, disposable containers, insulation materials, and consumer products. Consequently, although PS-based studies provide valuable mechanistic insights, caution should be exercised when extrapolating these findings to real-world exposure scenarios, where humans are simultaneously exposed to diverse polymer mixtures [29,32].

In this review, we have used the term MNP to collectively refer to micro- and nano-plastics.

### 2.2. Detection Studies in Reproductive Tissues

Building upon evidence of systemic human exposure, recent studies have increasingly focused on characterizing the distribution, abundance, and physicochemical characteristics of microplastics across different human tissues and biological matrices [18,33]. In humans, MPs display considerable heterogeneity in terms of particle size, morphology, and polymer composition. MP distribution within the human body appears to be highly organ-specific rather than governed by a universal particle-size threshold. Recent multi-organ analyses have demonstrated substantial variation in both microplastic abundance and size distribution across tissues. For example, Cao et al. reported the highest microplastic burdens in the liver (65.28 ± 23.94 particles/g), small intestine (61.06 ± 25.25 particles/g), and kidneys (58.63 ± 16.50 particles/g), whereas distinct size profiles were observed among organs. Larger particles were predominantly detected in the lungs (56.80 ± 57.70 μm), likely reflecting inhalation-mediated exposure, while particles smaller than 10 μm were more prevalent in the liver and spleen, suggesting enhanced systemic transport and tissue penetration. Furthermore, approximately 70% of microplastics detected in brain tissue ranged between 20 and 50 μm, indicating organ-specific selective accumulation. These findings suggest that microplastic deposition is influenced not only by particle size but also by factors such as exposure route, tissue vascularization, physiological barriers, clearance mechanisms, and polymer characteristics [34]. Consequently, organ-specific accumulation patterns should be considered when interpreting exposure assessments and comparing findings across studies.

Detailed characterization of reproductive tissues have revealed considerable variability in MP abundance, particle size, morphology, and polymer composition, highlighting the need to understand tissue-specific exposure patterns and potential biological implications [14,15,35]. Accordingly, several studies have specifically investigated their presence within the human reproductive system (Table 1).

Evidence from multiple studies consistently demonstrates the presence of microplastics in male reproductive samples, particularly semen and testicular tissue, indicating direct exposure of the male reproductive system. Reported findings include detection of a wide range of polymer types and particle sizes within these biological matrices. For instance, Montano et al. (2023) identified 16 different microplastic particles in semen samples from 10 participants, with sizes ranging from 2 to 6 µm, predominantly composed of PP, PS, and PET [14]. Similarly, Zhao et al. (2023) reported mean concentrations of 0.23 ± 0.45 MPs/mL in semen and 11.60 ± 15.52 MPs/g in testicular tissue, with PE and PVC dominating semen samples, while PS was most prevalent in testicular tissue [36]. Extending these findings, a larger cohort study involving 113 individuals detected microplastics in all semen and urine samples analyzed, with PS, PP, and PE identified as the most common polymers [37]. More recently, Guo et al. (2025) used laser direct infrared microscopy to identify 15 different types of MPs in 34 out of 45 semen samples and further reported a potential association between PET exposure and reduced sperm motility, suggesting possible functional reproductive toxicity [38].

Similarly, multiple studies have confirmed the presence of microplastics in female reproductive system samples, including reproductive fluids and reproductive tract tissues, indicating widespread exposure and tissue-level accumulation. In reproductive fluids, cervicovaginal lavage samples have shown measurable MP burden, with reported concentrations of 9.10 ± 14.96 MPs/g, predominantly comprising PP and PS particles smaller than 50 µm [39]. Similarly, follicular fluid has been identified as another key exposure site, with PE emerging as the dominant polymer and average concentrations reaching 8.8 ± 6.14 MPs/mL, suggesting direct interaction with the ovarian microenvironment [40]. At the tissue level, microplastics have been detected in multiple reproductive structures, including adenomyosis lesions, ovarian ectopic cysts, and fallopian tube tissues. Reported concentrations in these sites range from 1.4 ± 1.11 to 1.65 ± 1.31 MPs/g, with particle sizes generally below 20 µm and PE consistently present across all examined tissues [18]. Additional studies have further identified PS as the predominant polymer in endometrial polyp tissues, reaching concentrations as high as 94.81 ± 10.67 µg/g [41], while uterine fibroids and adjacent myometrium show comparatively lower (2.13 ± 1.17 and 0.88 ± 0.78 MPs/g, respectively) but detectable levels of MPs [42]. Furthermore, the detection of microplastics in both breast milk and placental tissues has raised concerns regarding their potential maternal–fetal transfer. In breast milk samples from 34 women, an average concentration of 0.52 MPs/g was reported [43]. Another study analyzing 62 placental samples found a substantially higher burden, with concentrations reaching up to 685 µg/g, with mean abundance being 128 ± 145 µg/g [13]. Across these studies, PE, PP, and PVC were the most frequently identified polymers, reinforcing concerns about their ability to cross biological barriers and potentially impact fetal development.

Despite the growing number of studies reporting microplastic occurrence in reproductive tissues, considerable variability exists in the reported concentrations, particle-size distributions, and polymer compositions. Such differences may reflect variations in geographic exposure patterns and biological factors, but may also arise from differences in sampling strategies, tissue processing methods, contamination-control procedures, and analytical approaches employed across studies [44]. Consequently, direct comparison of microplastic burdens between studies should be interpreted with caution. A detailed discussion of the analytical techniques currently used for MP detection, together with their respective strengths and limitations, is presented in the subsequent section.

### 2.3. Present MP Detection Methods and Analytical Limits

The accurate identification and quantification of MNPs in biological tissues remain technically challenging due to their diverse physicochemical characteristics and the complexity of biological matrices. Several analytical approaches have been employed for MP detection, each possessing distinct advantages and limitations that can substantially influence reported findings. Among the most widely applied techniques, micro-Fourier transform infrared spectroscopy (µ-FTIR) enables polymer-specific identification through characteristic infrared absorption spectra and is particularly useful for the analysis of larger microplastic particles. However, its analytical sensitivity is generally restricted to particles exceeding approximately 10–20 µm, limiting its ability to detect smaller MPs and NPs [45]. Raman spectroscopy offers improved spatial resolution and can detect particles approaching 1 µm in size, making it valuable for investigating smaller plastic fragments within tissues. Nevertheless, fluorescence interference from biological samples, prolonged analysis times, and challenges associated with complex matrices may affect measurement accuracy and reproducibility [45]. Laser direct infrared (LDIR) imaging has emerged as a rapid and automated technique for microplastic identification. Compared with conventional Fourier transform infrared (FTIR) and Raman spectroscopy, LDIR substantially reduces analysis time, making it well suited for high-throughput analysis of large numbers of particles. It can identify particles as small as approximately 10 μm with improved wavelength accuracy, facilitating microplastic analysis across diverse environmental matrices. However, its relatively narrow spectral range may limit polymer discrimination in certain applications [46].

The analytical performance of spectroscopic methods is also influenced by their limits of detection (LOD) and limits of quantification (LOQ), which are method- and instrument-specific and vary depending on instrument configuration, polymer type, sample preparation, and biological matrix. For particle-number-based measurements, the minimum detectable particle size largely determines analytical sensitivity, whereas universally applicable concentration-based LOD and LOQ values are difficult to establish because they depend on the analytical platform, sample matrix, and experimental workflow. Consequently, reported concentrations (particles/g tissue) are highly dependent on the analytical method employed, making direct comparisons across studies difficult [47].

Thermo-analytical methods, particularly pyrolysis–gas chromatography–mass spectrometry (Py-GC/MS), have emerged as powerful tools for polymer characterization. Unlike spectroscopic approaches, Py-GC/MS thermally decomposes plastic particles into characteristic chemical fragments, thereby enabling highly sensitive mass-based identification and quantification of polymer types, including polymers present as NP-sized particles. A major advantage of this technique is its ability to analyze complex biological and environmental samples with high chemical specificity. Py-GC/MS reports polymer concentrations as mass (e.g., μg/g tissue) rather than particle number because the particles are destroyed during analysis. Consequently, unlike imaging- or spectroscopy-based techniques, it cannot determine particle abundance, size distribution, morphology, or tissue localization [48]. Therefore, mass-based results obtained using Py-GC/MS cannot be directly compared with particle-number-based measurements (e.g., particles/g tissue) generated by spectroscopic methods such as µ-FTIR or Raman microscopy [49].

To improve characterization of smaller particles, field-flow fractionation (FFF) coupled with detectors such as multi-angle light scattering (MALS) or dynamic light scattering (DLS) has been increasingly employed. These approaches allow efficient separation of particles according to their hydrodynamic properties and facilitate the analysis of submicron-sized MPs and NPs [50]. Despite their enhanced size resolution, the accuracy of these methods can be affected by particle aggregation and the presence of organic matter within samples, which may reduce separation efficiency and lead to underestimation of particle abundance or inaccurate particle-size characterization [51]. More recently, advanced techniques have been developed to overcome the limitations of conventional analytical platforms. Surface-enhanced Raman spectroscopy (SERS) significantly increases signal intensity through plasmonic enhancement, enabling highly sensitive detection of nanoplastics at very low concentrations [52]. Similarly, nanoelectromechanical system-based Fourier transform infrared spectroscopy (NEMS-FTIR) has demonstrated exceptional analytical sensitivity, achieving sub-nanogram detection limits for common polymers such as polystyrene (PS), polypropylene (PP), and polyvinyl chloride (PVC) [53]. Although these emerging technologies show considerable promise, their application remains largely restricted to specialized research settings and often require sophisticated instrumentation and extensive sample preparation.

Reliable detection of MNPs also depends on rigorous quality assurance and quality control (QA/QC) procedures throughout the analytical workflow. Results are commonly corrected using procedural blank measurements to account for background contamination introduced during sample processing. Procedural blanks are routinely included to monitor laboratory and airborne contamination, while the use of filtered reagents, glass or metal laboratory equipment, cotton laboratory clothing, and clean-air workspaces minimizes the introduction of extraneous plastic particles during sample processing [54]. For spectroscopic techniques, polymer identification typically relies on spectral-library matching using predefined similarity thresholds established by individual laboratories or software packages to reduce false-positive identifications [55].

Additionally, spike-recovery experiments are commonly performed to evaluate analytical recovery and the efficiency of sample preparation, tissue digestion, and extraction procedures by spiking samples with known quantities of reference polymers. Recovery efficiencies vary considerably among analytical methods, polymer types, and biological matrices and should therefore be validated for each analytical workflow [56]. Moreover, complex biological matrices may interfere with particle isolation, polymer identification, and signal detection. Additionally, incomplete tissue digestion, particle loss during sample preparation, particle aggregation, or stringent spectral-matching criteria may contribute to underestimation of MNP abundance, whereas laboratory contamination, airborne particles, or misidentification of non-plastic materials may lead to overestimation if appropriate QA/QC measures are not implemented. These quality-control measures improve analytical reliability and reproducibility but are not yet standardized across laboratories [51].

Despite substantial technological progress, several methodological challenges continue to hinder the reliable assessment of MNP occurrence in human tissues. Variations in sample collection, tissue digestion protocols, contamination-control measures, procedural blank correction, particle isolation methods, analytical detection limits, polymer identification criteria, QA/QC practices, and the reporting of either particle-number-based (particles/g) or mass-based (μg/g) measurements contribute to considerable heterogeneity among studies and complicate direct comparisons of reported findings. Consequently, the lack of standardized analytical workflows, harmonized QA/QC procedures, and reporting guidelines remains a major obstacle to accurately assess MNP occurrence in human tissues and comparing results across different investigations.

**Table 1:** Occurrence and characterisation of microplastics in human samples pertaining to reproductive systems.

Sample	Study Population	Detection Method	Characteristics	Predominant Polymers	Biological Implications	References
Semen	Multiple cohorts	µ-Raman, PY-GC/MS, LD-IR	Distinct types of MPs detected; size range: 2–100 µm; fragment-shaped	PP, PS, PE, PET, PVC	Associated with reduced sperm quality, including motility and concentration	[14,36,38]
Testicular tissue	Small cohort studies	PY-GC/MS	Size range: 20–100 µm; fragment shaped	PS	Limited functional data	[36]
Cervicovaginal fluid	Small cohort	µ-Raman	Size < 50 µm; fragment shaped	PP, PS	Possible link to usage of menstrual devices	[39]
Reproductive tissues (adenomyosis, fibroids, uterus)	Clinical samples	µ-Raman, PY-GC/MS	Adenomyosis- Size < 20 µm; Fiber/Film shaped Myometrium- size: 1–497 μm Irregular shapes	PE, PP, PS, PVC, PET	Correlations with age, BMI, and disease characteristics	[18,41,42]
Follicular fluid	IVF patients	µ-Raman, microscopy	Size < 10 µm	PE, PP, PET	Negative association with fertilization rate	[15,40]
Breast milk	Lactating women	µ-Raman	Size: 2–12 µm; Irregular fragments and spheres	PE, PP, PVC	MPs have the capacity to accumulate and carry bacteria	[43]
Placenta	Pregnant women (hospital-based cohort)	µ-Raman	Size: 5–10 µm; Irregular shape, fibres and spheres	Various	Found in the fetal side, maternal side and chorioamniotic membranes	[13,57]

## 3. Potential Mechanisms Linking Microplastic Exposure to Cancer-Related Processes

One proposed mechanism by which microplastics may influence cancer-related biological processes involves disruption of cellular membrane integrity following particle internalization, leading to increased intracellular reactive oxygen species (ROS) production, oxidative stress, mitochondrial dysfunction, and inflammatory signaling [10,58,59]. Elevated ROS levels following MP exposure have also been associated with alterations in cellular death pathways through multiple mechanisms [9]. With regard to pyroptosis, MPs have been shown to activate the NLRP3 inflammasome, leading to caspase-1 activation, cleavage of gasdermins D and E, and subsequent formation of membrane pores that facilitate the release of pro-inflammatory cytokines such as IL-1β and IL-18 in ovarian granulosa cells [9]. Similarly, dysregulation of ferroptosis-related pathways has been observed following MP exposure in several experimental models, including prostate-derived cells [23,60]. While these responses may contribute to pathways associated with carcinogenesis, their occurrence alone is insufficient to infer a causal relationship between microplastic exposure and cancer development.

In addition to affecting cell death pathways, MPs have been shown to promote genotoxicity primarily through oxidative stress [10]. Elevated ROS generated upon exposure directly damage DNA, leading to strand breaks, base alterations, and adduct formation, while impairing key repair pathways, including base excision repair and homologous recombination, contribute towards chromosomal instability [61]. Moreover, MPs can act synergistically with co-contaminants like arsenic, phthalates, etc., intensifying these effects [25]. However, the extent to which these molecular alterations translate into increased cancer risk under real-world exposure conditions remains unclear.

Experimental evidence also suggests that MPs may influence cellular behaviors commonly associated with malignant phenotypes. For instance, PP-MPs have been reported to upregulate the expression of genes such as transmembrane BAX inhibitor motif-containing 6 (TMBIM6) and adaptor-related protein complex 2 subunit mu 1 (AP2M1), which are involved in signaling pathways relevant to cell survival and migration. Notably, TMBIM6 is known to activate the Mechanistic Target of Rapamycin Complex 2 mTORC2/AKT signaling pathway, which plays a critical role in promoting metastatic behavior [62]. Similarly, exposure to PS-NPs has been reported to increase the expression of stemness-related markers such as CD44 and Aldehyde dehydrogenase 1 (ALDH1), thereby promoting the expansion of less differentiated cell populations with enhanced migratory and invasive potential [63]. Overall, MPs seem to promote tumor development by creating an oxidative stress–driven link between programmed cell death and DNA damage. However, most findings are based on in vitro studies. The long-term in vivo effects, precise mechanisms of genotoxicity, and differences in carcinogenic potential across MPs with varying physicochemical characteristics are still unclear and require well-designed animal and epidemiological research.

## 4. Male Reproductive System Cancers: Association with Microplastic Exposure

### 4.1. Prostate Cancer

Prostate cancer ranks as the second most (after lung cancer) commonly diagnosed cancer in men and is the fifth leading cause of cancer-related mortality worldwide. Although largely driven by hormonal regulation and androgen signaling, recent findings have drawn attention to the potential involvement of microplastics. Multiple studies have reported the detection of MPs in prostate tissue utilizing different methods with particle sizes ranging from 1 to 100 micrometers and various types and shapes including pellets, spheres, and fibers (Table 2) [64,65,66].

A recent study reported that exposure to PS-MPs (0.1 μg/mL) induced mild oxidative stress in prostate cancer cells (LNCaP), thus promoting proliferation. Further analysis revealed significant changes in the expression of inflammatory cytokines following PS exposure. Notably, levels of eotaxin, interferon-gamma (IFN-γ), and interleukin-12 (IL-12) were decreased, whereas interleukin-4 (IL-4) was increased. Importantly, the study also identified ferroptosis, an iron-dependent form of cell death, as a key pathway affected by PS exposure. Specifically, PS promoted the expression of glutathione peroxidase 4 (GPX4), an enzyme that enhances cellular antioxidant defenses. By upregulating GPX4, PS suppressed ferroptosis, allowing cancer cells to survive and proliferate [23]. Building on these findings, another study further demonstrated that PS particles significantly enhanced not only proliferation but also migration and invasion of prostate cancer cells through ferroptosis regulation.

**Figure 2:**
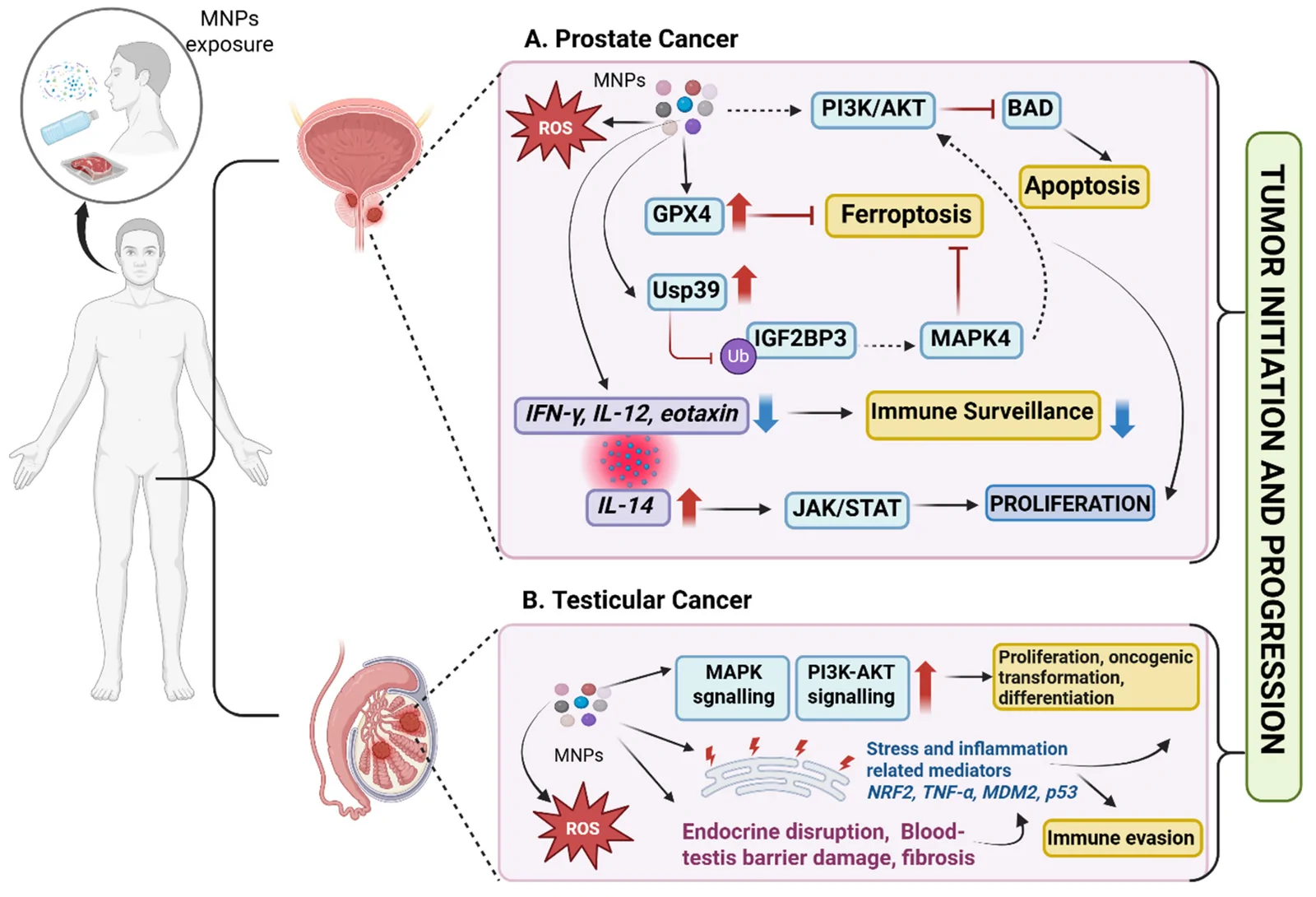
Potential mechanisms by which micro- and nanoplastics (MNPs) may contribute to processes associated with male reproductive cancers. (**A**) In prostate cancer, MNP exposure has been associated with increased reactive oxygen species (ROS) generation, oxidative stress, dysregulation of ferroptosis- and apoptosis-related pathways (including PI3K/AKT, BAD, GPX4, USP39, IGF2BP3, and MAPK4), and modulation of inflammatory cytokines and immune mediators such as IFN-γ, IL-12, eotaxin, and IL-14. These alterations may influence immune surveillance, activate JAK/STAT signaling, and create cellular conditions that are permissive for tumor progression. (**B**) In the testis, experimental studies suggest that MNP exposure may alter MAPK and PI3K/AKT signaling, increase oxidative stress and inflammatory responses, and disrupt endocrine homeostasis. Collectively, these changes may affect cellular proliferation, differentiation, and other processes associated with carcinogenesis. Created in BioRender. Afroz, N. (2026). https://BioRender.com/b5kptb1. (accessed on 10 May 2026).

This study identified a molecular mechanism through which PS exposure suppresses ferroptosis and enhances cancer progression. PS-induced upregulation of Ubiquitin-specific peptidase 39 (USP39), a spliceosome-associated protein increasingly recognized for its oncogenic role in multiple cancers [67]; leading to stabilization of Insulin-like growth factor 2 mRNA-binding protein 3 (IGF2BP3) by inhibiting its ubiquitin-mediated proteasomal degradation and allowing it to bind and protect Mitogen-Activated Protein Kinase 4 (MAPK4) mRNA from degradation, resulting in elevated MAPK4 expression. Increased MAPK4 activates pro-survival pathways, particularly AKT/mTOR signaling, along with others that reduce lipid peroxidation and oxidative membrane damage, thereby inhibiting ferroptosis cell death [68]. Consequently, cancer cells develop greater resistance to oxidative stress along with enhanced proliferative, migratory and invasive capabilities. This study uncovered a distinct molecular mechanism by which PS exposure modulates ferroptosis in a manner that promotes more aggressive cancer cell behavior, including increased motility and invasiveness [24] (Figure 2).

Building on these insights, the ferroptosis pathway may offer potential biomarkers for early detection and therapeutic targets to mitigate microplastic-related prostate cancer risk.

Alongside MPs, exposure to BPA has also been linked to prostate cancer via multiple overlapping mechanisms [69]. Neonatal exposure to both low and high doses of BPA induces carcinogenic changes in prostate cells, including altered cell morphology, disrupted gene expression, and epigenetic modifications such as hypomethylation of the nucleosome binding protein-1 promoter [70]. BPA also binds to the androgen receptor, interfering with key molecular processes including the silencing of phosphodiesterase type 4 (a critical metabolic enzyme) and altering DNA methyltransferases, thereby promoting the formation of neoplastic lesions and activating oncogenes, collectively creating a predisposing environment for prostate cancer development.

**Table 2:** Microplastics and Male & Female Reproductive Cancers—Experimental Evidence.

Primary Contact Tissue	Experimental Models	MNPs (Type, Size, Dose)	Key Effects	Key Mechanisms	Ref.
Prostate	In vitro (LNCaP prostate cancer cells)	Type: PS-MPs; Dose: 0.1 µg/mL	Increased proliferation; altered cytokine expression; enhanced migration and invasion	Oxidative stress; ferroptosis inhibition via GPX4; USP39–IGF2BP3–MAPK4 axis	[23,24]
	Human tissue studies	Various polymers (PE, PP, PS); Size: 1–100 µm	Presence and accumulation of MPs in prostate tissue	—	[64,65,66]
Penile tissue	Human samples (PeC patients)	Type: PE, PP, PVC; Size: 20–50 µm	Higher MP load and diversity in tumor vs. normal tissue	Potential tumor microenvironment modulation	[71]
Testis	Microfluidic organ-on-chip (HK-2, NTERA-2, HUVEC)	Type: PS-NPs; Size: 50 nm	Cellular ultrastructure disruption; activation of tumor-related pathways	MAPK and PI3K/AKT activation; NRF2, TNF-α signaling; apoptosis regulators (p53, MDM2, BAD)	[72]
	Epidemiological studies	Type: PVC (occupational exposure)	Increased seminoma risk (inconsistent findings)	—	[73]
Ovary	In vivo (mouse model) + in vitro (HEY cells)	Type: PS-NPs; Size: 100 nm; Dose: 10 mg/L	Accelerated tumor growth (in vivo); variable proliferation (in vitro)	Tumor microenvironment modulation; thrombomodulin pathway	[74]
	In vitro + in vivo	Type: PS-NPs; Size: 50 nm	Increased proliferation	CDK4/6 pathway activation	[75]
	In vitro (granulosa cells)	Type: PS-MPs + DEHP	Oxidative stress; DNA damage; cell injury	ROS generation; Hippo signaling activation	[25]
	In vivo	Type: PS-MPs; Size: 5 µm	Ovarian toxicity; biomarker alterations	Oxidative stress; cytoskeletal disruption	[76]
	In vitro + in vivo (Human ovarian granulosa cells and female rat)	Type: PS-NPs; Dose: 100 µg/mL	Decreased viability; apoptosis	Oxidative stress; CTGF, Cyr61 dysregulation	[77]
	In vivo	0.5 µm PS-MPs;	Oxidative stress	Inhibition of Keap1/Nrf2/HO-1 signaling pathway	[78]
Endometrium	In vitro (3D model)	MPs; Dose: 10–50 mg/mL	Barrier disruption; fibrosis	Reduced ZO1/CDH1; pro-fibrotic signaling	[79]
	In vitro + in vivo	Type: PVC-MPs	Increased proliferation, migration, invasion	AHR/CYP1A1 pathway activation	[80]
	In vitro (HEC-1B, organoids)	Type: PS-NPs; Size: 100 nm	Tumor progression; EMT	AMPK–ACSS2 axis; histone acetylation; PLA2G3 activation	[81]
Cervix	Human samples	Type: PE, PP; Size: <50 µm	Higher MP levels in cancer tissue; increased tumor aggressiveness	Metabolic reprogramming (carbohydrate metabolism)	[6,82]
Vaginal tissue	In vitro (keratinocytes)	Type: PE; Size: 200 nm–9 µm	Cytotoxicity; structural damage; epigenetic alterations	Oxidative stress; miRNA dysregulation; DNA methylation changes	[83]

### 4.2. Penile Cancer

As a rare malignancy, penile cancer (PeC) accounts for 36,068 cases worldwide, placing it 30th on the global list of the most frequently diagnosed cancers, with more prevalence in developing countries [84]. Although rare, it is highly aggressive, affecting the skin of the glans or inner foreskin, marked by invasive growth and early lymph node metastasis. Its treatment often causes significant functional and cosmetic impairments, strongly impacting patients’ quality of life [85].

A 2025 study provides the first direct evidence connecting microplastic accumulation to penile cancer in humans. Tissue samples from 17 PeC patients were evaluated using Laser Direct Infrared (LDIR) spectroscopy, allowing the researchers to compare cancerous tissue with adjacent non-cancerous tissue. The results aligned with findings from other tissue studies, where malignant tissues typically contain higher microplastic load and diversity than nearby normal tissues. PE, PP, and PVC were the main polymers detected, mostly falling in the 20–50 μm size range [71]. These findings suggest that MPs may play an active role in the tumor microenvironment or even in the carcinogenic progression of PeC. However, given the preliminary nature of these findings, substantial research is needed to establish causality and elucidate the underlying mechanisms.

### 4.3. Testicular Cancer

Testicular cancer (TCa) is rare in both incidence and mortality, yet it poses a substantial clinical challenge due to its adverse effects on male reproductive health [86]. Although generally not classified as an occupational disease, industrial exposure within plastic-related sectors, specifically the production and manufacturing of PVC, has been associated with an increased risk of testicular cancer but evidence on this claim remains inconsistent [87,88].

One case–control study reported a marked increase in seminoma risk (up to sixfold) with PVC exposure, though findings varied depending on pre-existing conditions like orchitis or cryptorchidism, and no clear association was seen with other plastics [73]. Moreover, cohort studies on vinyl chloride and other plastics found no significant rise in testicular cancer risk. Some discrepancies may be due to exposure misclassification, suggesting that only workers with direct and substantial contact with plastic compounds should be assessed [87,89,90]. While direct epidemiological evidence remains inconclusive, numerous experimental studies have shown that MPs/NPs can induce oxidative stress, inflammation, endocrine disruption, and blood–testis barrier damage, thereby creating conditions favorable for testicular carcinogenesis [91]. Additionally, MPs act as carriers for endocrine-disrupting chemicals (EDCs), which are known to contribute to TCa by interfering with hormonal regulation, inducing cellular proliferation, and promoting tumor-associated signaling pathways in testicular cells [12,92].

Emerging mechanistic evidence supports the potential role of micro- and nanoplastics (MNPs) in TCa pathogenesis. A study utilized a bionic kidney–testis microfluidic platform to simulate inter-organ interactions and evaluate the toxicity of PS-NPs (50 nm). In this system, human renal epithelial cells (HK-2) and testicular cancer cells (NTERA-2 cL. D1) were co-cultured with endothelial cells to mimic physiological connectivity, revealing that PS-NPs were internalized via endocytic vesicles and subsequently disrupted cellular ultrastructure following short-term exposure. These alterations were accompanied by dysregulation of key oncogenic signaling pathways, particularly the MAPK and PI3K/AKT axes, which are critically involved in cell proliferation, survival, and tumor progression. Additionally, activation of stress- and inflammation-related mediators, including Nuclear factor erythroid 2-related factor 2 (NRF2), Tumor necrosis factor-alpha (TNF-α) signaling, and regulators of apoptosis such as p53 and mouse double minute 2 (MDM2), further highlights the multifaceted impact of MNPs on tumor-promoting pathways [22,72,93] (Figure 2). Although direct clinical evidence remains limited, these mechanistic insights underscore the potential of MP exposure to disrupt key molecular processes within testes and may influence processes associated with oncogenic transformation.

## 5. Female Reproductive Cancers: Association with Microplastic Exposure

### 5.1. Ovarian Cancer

Ovarian cancer remains one of the most lethal gynecological malignancies, ranking among the leading causes of cancer-related incidence and mortality in women worldwide [94,95]. Emerging evidence suggests that microplastics (MPs), particularly PS-MPs/NPs, may influence ovarian tumor biology in complex and sometimes contrasting ways depending upon particle size, concentration, exposure duration, and the presence of co-contaminants (Table 2) [96]. Recent experimental work has demonstrated that chronic exposure to PS-MPs can modulate ovarian cancer progression through alterations in the tumor microenvironment (TME). For instance, in vivo studies using mouse models have shown that administration of nanosized PS particles (100 nm, 10 mg/L) accelerates epithelial ovarian cancer (EOC) tumor growth. Interestingly, parallel in vitro experiments using HEY ovarian cancer cell lines reported a dose-dependent inhibitory effect on cell proliferation, highlighting context-dependent responses to MP exposure [74]. Mechanistically, these effects appear to be mediated through changes in gene expression linked to thrombomodulin and its regulatory network, which are known to influence immune signaling and metabolic pathways within the tumor microenvironment [97,98]. Bioinformatic analyses of ovarian cancer datasets (TCGA-OV) further support the involvement of thrombomodulin-associated pathways as potential mediators of MP-induced tumor modulation [74]. These changes may create a tumor microenvironment marked by hypoxia, pH imbalance, and oxidative stress, all linked to aggressive tumor behavior [99].

**Figure 3:**
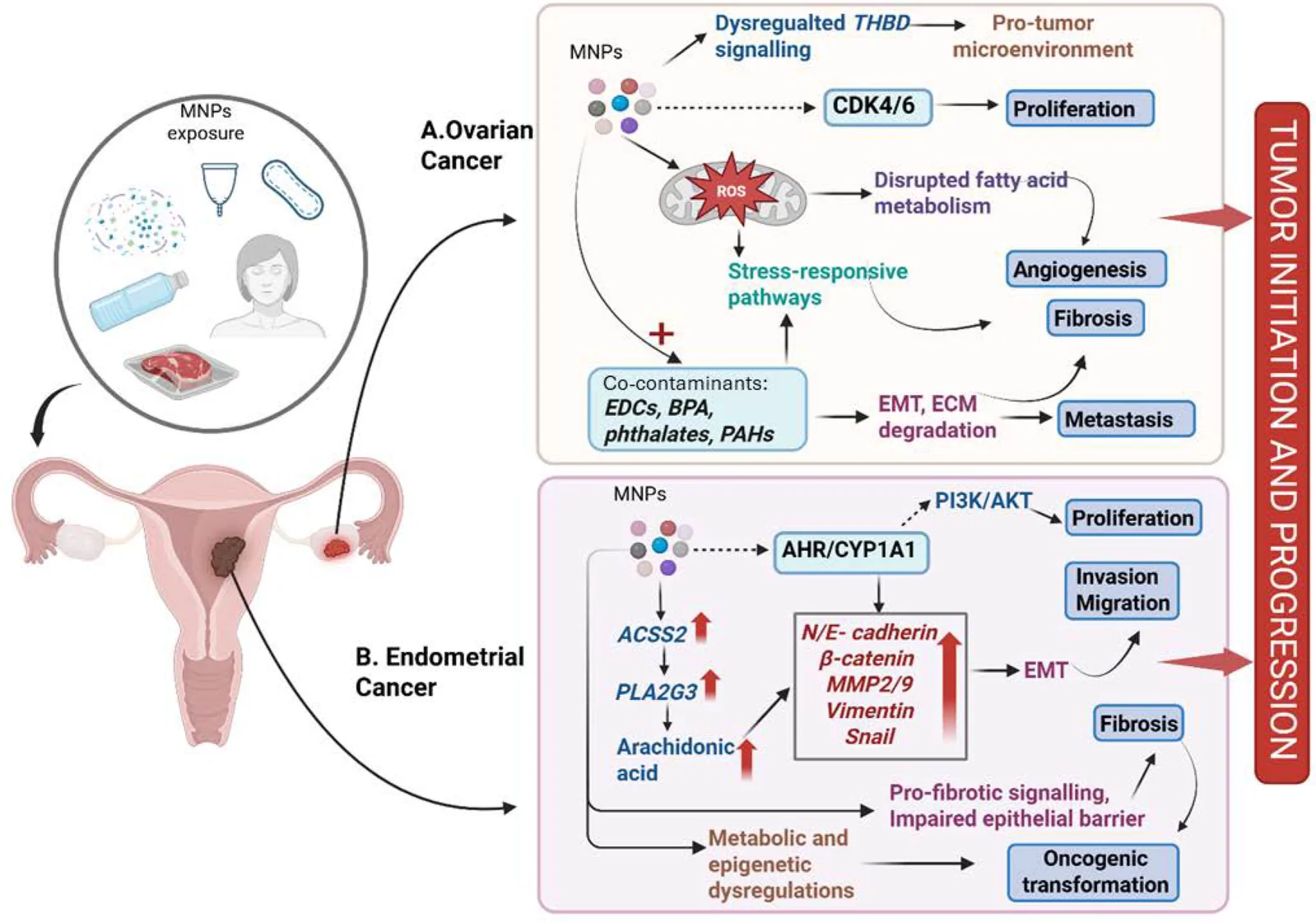
Potential mechanisms by which MNPs may influence biological processes associated with female reproductive cancers. (**A**) In ovarian cancer, MNP exposure has been associated with increased reactive oxygen species (ROS) generation, mitochondrial dysfunction, activation of stress-responsive pathways, and alterations in fatty acid metabolism. Experimental findings also suggest that MNP exposure may influence THBD signaling and CDK4/6-related proliferative pathways. In addition, MNPs can act as carriers of co-contaminants, including endocrine-disrupting chemicals (EDCs), bisphenol A (BPA), phthalates, and polycyclic aromatic hydrocarbons (PAHs). Collectively, these alterations may influence oxidative stress, extracellular matrix (ECM) remodeling, epithelial–mesenchymal transition (EMT), angiogenesis, fibrosis, and other biological processes that have been implicated in fostering a pro-tumorigenic environment. (**B**) In endometrial cancer, experimental studies suggest that MNP exposure may influence AHR/CYP1A1 and PI3K/AKT signaling pathways and alter lipid metabolism through changes in ACSS2, PLA2G3, and arachidonic acid production. These alterations have been associated with increased expression of EMT- and metastasis-related markers, including N-cadherin, β-catenin, MMP2/9, vimentin, and Snail. MNP exposure has also been associated with metabolic and epigenetic alterations that may affect epithelial barrier integrity, pro-fibrotic signaling, cellular invasion and migration, and other pathways relevant to oncogenic transformation. Created in BioRender. Afroz, N. (2026). https://BioRender.com/4tkcv0t. (accessed on 8 May 2026).

Furthermore, exposure to PS-MPs has been associated with elevated oxidative stress in ovarian tissues, a condition strongly linked to tumor initiation, progression, and metastasis. Such oxidative imbalance may therefore create a favorable microenvironment for ovarian carcinogenesis. One of the major molecular targets affected by PS-MPs is the Kelch-like ECH-associated protein 1 (Keap1)–Nuclear factor erythroid 2–related factor 2 (Nrf2) signaling pathway, a critical regulatory system which normally functions to maintain cellular redox homeostasis and defend cells against oxidative injury. Interference with this antioxidant defense mechanism by MPs results in excessive ROS accumulation, leading to DNA instability, biomolecular damage, and activation of pathways involved in malignant transformation [78]. Additionally, exposure to low concentrations of PS-NPs (50nm) has been shown to enhance ovarian cancer cell proliferation in both cellular and animal model. These particles are readily internalized by cancer cells, promoting cell cycle progression via a CDK4/6-dependent mechanism (Figure 3). Notably, this proliferative effect is effectively reversed by the CDK4/6 inhibitor Palbociclib, a finding that has also been confirmed in in vivo models [75].

In addition to direct effects, MPs exposure leads to significant toxicity in ovarian tissue, which may influence processes associated with oncogenic transformation. Studies examining combined exposure to PS-MPs and the plasticizer di-(2-ethylhexyl) phthalate (DEHP) have demonstrated synergistic toxicity in ovarian granulosa cells. This interaction leads to elevated reactive oxygen species (ROS) generation, oxidative DNA damage, and activation of stress-responsive pathways such as the Hippo signaling cascade, which has been linked to cell cycle arrest, cellular injury, and necroptotic cell death [25]. Additionally, animal studies indicate that exposure to PS-MPs disrupts normal ovarian physiology. PS-MPs of 5 µm in size have been associated with oxidative stress-induced alterations in ovarian biomarkers and cytoskeletal protein expression, suggesting broader reproductive toxicity that could increase susceptibility to malignant transformation [100]. Contrastingly, in several models, MP-induced oxidative stress and DNA damage appear sufficient to trigger cytotoxic responses that suppress cancer cell survival [76]. For instance, studies on granulosa-like tumor cell models have demonstrated that accumulation of PS nanoplastics (100 μ g/mL) resulted in decreased cell viability, increased oxidative stress, and induction of apoptosis alongside dysregulation of downstream targets such as Connective tissue growth factor (CTGF) and Cysteine-rich angiogenic inducer 61 (Cyr61) which have been shown to promote cancer progression by enhancing cell proliferation, angiogenesis and remodelling extracellular matrix and tumor immune microenvironment [77,101,102]. Besides cytotoxic effects, microplastics have been shown to disrupt ovarian function through metabolic and stress-related pathways. In animal models, administration of PS-MPs (30 mg/kg) impaired oocyte maturation, increased oxidative stress, and disrupted fatty acid metabolism, along with activation of endoplasmic reticulum (ER) stress and gene expression changes associated with and ovarian aging, fibrosis and angiogenesis [103].

Beyond direct effects, MPs may enhance ovarian carcinogenesis by serving as carriers for EDCs, including Bisphenol A (BPA) and benzophenones, which are known to stimulate proliferative signaling and tumor progression in ovarian cells [12,104]. BPA has been shown to potentiate growth and invasiveness of ovarian cancer cells through multiple converging mechanisms. For example, BPA suppressed apoptotic pathways, including downregulation of key pro-apoptotic genes, enhancing epithelial–mesenchymal transition, evidenced by an increased N-cadherin-to-E-cadherin ratio and upregulating the expression of matrix metalloproteinases (MMP-2 and MMP-9), facilitating extracellular matrix degradation and cancer cell invasion [105,106,107]. Additionally, BPA promotes microenvironmental remodelling by activating the Wnt/β-catenin signaling pathway, leading to Secreted Phosphoprotein 1 (SPP1)-driven osteopontin secretion and subsequent transformation of fibroblasts into cancer-associated fibroblasts, thereby driving tumor microenvironment remodelling, angiogenesis and immune evasion supporting metastatic progression [107,108].

Taken together, these findings highlight a multifaceted role of microplastics in ovarian carcinogenesis, involving direct modulation of tumor cell behavior, reprogramming of the tumor microenvironment, and interaction with co-existing environmental toxicants.

### 5.2. Endometrial Cancer

Endometrial cancer (EC), a hormone-driven malignancy, is strongly associated with unopposed estrogen exposure and metabolic dysfunction. While traditional risk factors such as obesity and endocrine imbalance are well established, environmental contaminants like microplastics are increasingly being recognized as potential modulators of disease progression [109]. Recent studies have demonstrated the presence of MPs within endometrial tissues, including both normal endometrium and pathological conditions such as endometrial polyps, where their abundance is significantly higher. The predominant detection of polymers such as PE and PP in EC tissues further supports their accumulation in the uterine microenvironment [41]. Animal studies have demonstrated that MPs can infiltrate uterine tissues through multiple pathways, including systemic transport via blood circulation for smaller particles and direct vaginal–uterine translocation for larger particles up to 100 μm in size [110]. In a 3D human endometrial in vitro model, high-dose MP exposure (10–50 mg/mL) has been shown to impair the endometrial epithelial barrier, reduce junctional protein expression (e.g., ZO1 and CDH1), and activate pro-fibrotic pathways leading to increased collagen deposition, suggesting early endometrial remodeling events that may predispose the tissue to pathological changes [79].

A recent investigation demonstrated that PVC-MPs promote endometrial cancer progression by enhancing cellular proliferation, migration, and invasion through activation of the Aryl Hydrocarbon Receptor (AHR)-cytochrome P450 1A1 CYP1A1 signaling pathway. This pathway, known to regulate xenobiotic metabolism and mediate cellular responses to environmental toxins, plays a critical role in driving tumor-promoting processes [111,112]. Importantly, these effects were consistently validated in both in vitro and in vivo systems [80]. In addition to signaling alterations, metabolomic analyses indicate that MP exposure is linked to significant disruptions in cancer-associated metabolic pathways, particularly glycine, serine, and threonine metabolism. Notable changes were observed in metabolites such as glycine, N-acetyl-arginine, and 4-aminobutyric acid, which are known to influence tumor growth and immune responses [113]. Furthermore, PS-NPs have been shown to be internalized and accumulate within HEC-1B cells and EC organoids, where they promote tumor progression.

Mechanistically, exposure to 100 nm PS-NPs induces metabolic and epigenetic reprogramming in EC cells via activation of the AMP-activated protein kinase–Acyl-CoA synthetase short-chain family member 2 (AMPK–ACSS2) signaling axis, which facilitates the nuclear translocation of ACSS2, where it contributes to increased histone H3 H3K9 acetylation. This in turn upregulates phospholipase A2 group III (PLA2G3) expression, leading to enhanced arachidonic acid production and altered lipid metabolism [114]. Elevated arachidonic acid levels further promote inflammatory and pro-tumorigenic signaling, ultimately driving epithelial–mesenchymal transition (EMT), a process characterized by loss of epithelial features and acquisition of migratory and invasive properties that facilitate cancer progression and metastasis in EC cells [81,115].

Collectively, these findings suggest that microplastics contribute to endometrial carcinogenesis through a combination of tissue accumulation, metabolic disruption, and activation of oncogenic signaling pathways (Figure 3). Building on these insights, these pathways may serve as potential biomarkers for early detection, targets for therapeutic intervention, and help limit microplastic-related risk in endometrial cancer.

### 5.3. Cervical Cancer

Cervical cancer is ranked as the fourth most common cancer among women globally [116]. The first comprehensive detection of microplastics in human cervical cancer tissues was achieved using advanced Raman spectroscopy in a study by Xu et al. Thirteen different MPs were identified in blood and cervical cancer samples, among which PE, PP and polyethylene-co-polypropylene were the most abundant. MP levels were significantly higher in malignant tissues than in adjacent normal tissues, with blood samples displaying the greatest abundance and diversity of particles, evidence of systemic distribution [117].

Particles under 50 μm dominated all samples, indicating efficient tissue penetration. Frequent consumption of bottled water and plastic-bottled beverages correlated with elevated MP levels, highlighting dietary intake as a key exposure pathway. However, these observations do not establish a direct relationship between bottled beverage consumption and cervical cancer development, and further studies are needed to determine whether increased microplastic exposure contributes to disease risk or progression. [6,118]. Furthermore, a subsequent study by the same team reported that invasive cancers contained significantly more microplastic particles than early-stage lesions. A deeper metabolic analysis of these tissues revealed that tumors with high microplastic loads had disrupted carbohydrate metabolism, particularly involving D-mannose and the pathways governing amino sugars and nucleotide sugars. Such metabolic disruptions suggest that microplastics may actively remodel the tumor microenvironment, potentially fueling the metabolic reprogramming that enables cancers to become more aggressive [112,119]. Collectively, the evidence positions cervical cancer as a notable site of microplastic accumulation, raising the possibility that MPs contribute to both disease onset and progression. The metabolic signatures uncovered in these studies offer preliminary insights into novel mechanisms by which microplastics could influence cancer biology. Nonetheless, establishing causality and understanding the long-term health consequences of MP exposure in cervical cancer will require further investigation.

### 5.4. Vaginal Cancer

Primary vaginal cancer is rare, accounting for only 1–2% of female reproductive tract malignancies, with high prevalence particularly in areas with a lower human development index (HDI) and a high incidence of human papillomavirus (HPV) infection. Most vaginal lesions are metastatic, typically originating from nearby reproductive organs (cervix, endometrium, ovary) or distant sites (colon, breast, pancreas) [120,121].

Evidence regarding the carcinogenic potential of MPs in vaginal tissues is currently limited, but in vitro studies suggest multiple biological pathways that could contribute to cellular dysfunction and oncogenic transformation. In vaginal keratinocytes exposed to PE particles (200 nm–9 μm), acute high-dose exposure induced cytotoxicity, structural damage and disrupted oxidative stress–related gene expression, along with miRNAs involved in epithelial barrier regulation. Although partial recovery in viability and differentiation/proliferation markers was observed after exposure cessation or during chronic exposure, persistent alterations in DNA methyltransferase and demethylase expression were detected, suggesting potential epigenetic dysregulation, which may contribute to long-term processes such as inflammation, accelerated cellular aging, or malignant transformation [22,83]. These preliminary findings warrant further studies.

## 6. Critical Discussion of Current Evidence

Although numerous studies have reported the presence of microplastics in reproductive tissues and have demonstrated biological effects relevant to carcinogenesis, important inconsistencies remain across the literature. Variations in reported microplastic abundance, polymer composition, and particle size distributions are frequently observed among studies examining similar tissues. These discrepancies may reflect differences in geographic exposure patterns, demographic characteristics of study populations, sample sizes, and analytical methodologies. In particular, differences in detection sensitivity and contamination-control procedures can substantially influence reported microplastic concentrations.

Similarly, mechanistic studies have not always produced consistent findings. While oxidative stress, inflammatory responses, endocrine disruption, and alterations in cell-signaling pathways are commonly reported following microplastic exposure, the magnitude and nature of these effects vary considerably depending on polymer type, particle size, surface characteristics, exposure duration, and experimental model. A notable limitation of the current evidence base is the predominance of PS in experimental studies. While PS provides a useful model polymer, environmental exposures involve heterogeneous mixtures of plastics with distinct physicochemical characteristics. Furthermore, several studies have reported higher microplastic burdens in diseased or tumor tissues compared with adjacent non-cancerous tissues. However, the directionality of this association remains unclear. It is currently uncertain whether microplastic accumulation contributes to disease development or whether pathological alterations in tissues facilitate increased particle retention. Also, the absence of a clear increase in reproductive cancer incidence attributable to microplastic exposure may reflect the multifactorial nature of cancer development, long disease latency periods, challenges in exposure assessment, and the current lack of prospective epidemiological studies. Consequently, existing evidence should be interpreted as demonstrating associations rather than causality. Taken together, these limitations highlight the need for standardized methodologies, environmentally relevant exposure models, larger human cohorts, and longitudinal investigations capable of distinguishing correlation from causation and clarifying the role of microplastics in reproductive carcinogenesis.

## 7. Knowledge Gaps and Future Directions

Despite increasing evidence linking microplastics (MPs) to reproductive toxicity and cancer-related biological processes, substantial knowledge gaps remain. The lack of methodological standardization, variability in experimental designs, and limited human evidence continue to restrict robust risk assessment and causal inference. Future research should prioritize:

**Standardization of detection and exposure assessment:** Harmonized protocols for sample collection, processing, contamination control, and MP characterization are needed to improve comparability across studies and enable more reliable exposure assessment.

**Mechanistic and molecular characterization:** Future studies should identify molecular signatures associated with MP exposure, including alterations in gene expression, epigenetic regulation, and inflammatory pathways, to better understand their potential role in reproductive carcinogenesis.

**Human studies and advanced models:** Large-scale longitudinal studies are required to clarify causal relationships between MP exposure and reproductive cancers. In parallel, advanced models such as organoids and organ-on-chip systems, together with environmentally relevant exposure scenarios involving mixed polymers and aged particles, should be utilized to improve translational relevance.

## 8. Conclusions

Micro- and nanoplastics (MNPs) have become pervasive environmental contaminants and have been detected in a variety of human tissues, including reproductive organs. Growing evidence from experimental studies, animal models, and human tissue analyses suggests that MNP exposure may interact with biological processes relevant to cancer development, including oxidative stress, inflammation, endocrine disruption, genotoxicity, and alterations in cellular signaling pathways. However, the current body of evidence does not establish a direct causal relationship between MNP exposure and reproductive cancers. The available literature indicates that MNPs can modulate cellular responses and molecular pathways that have been implicated in carcinogenesis in other contexts. Nevertheless, most mechanistic findings originate from in vitro studies or animal models, frequently employing exposure concentrations, particle characteristics, and experimental conditions that may not accurately reflect real-world human exposure. Furthermore, the majority of human studies are observational or cross-sectional in nature and therefore cannot determine whether MNP accumulation contributes to cancer development or whether cancer-associated tissue alterations promote increased particle uptake and retention.

Interpretation of the current evidence is further complicated by substantial methodological variability among studies. Differences in sampling procedures, contamination control, particle extraction methods, analytical detection techniques, and reporting standards may influence estimates of microplastic abundance and limit direct comparisons across investigations. In addition, much of the existing toxicological literature relies on PS particles, whereas real-world environmental exposure involves complex mixtures of multiple polymer types. Taken together, current evidence supports the biological plausibility of a relationship between MNP exposure and reproductive cancer-related processes; however, significant knowledge gaps remain regarding exposure assessment, dose–response relationships, temporal associations, and causality. Future research should prioritize standardized analytical methodologies, environmentally relevant exposure models, prospective epidemiological studies, and advanced experimental platforms, including organoids and organ-on-chip systems. Such approaches will be essential for clarifying the significance of MNP accumulation in reproductive tissues, distinguishing association from causation, and improving our understanding of the potential health implications of long-term MNP exposure.

## Data Availability

No new data were created or analyzed in this study. Data sharing is not applicable to this article.
